# The effect of sex work regulation on health and well‐being of sex workers: Evidence from Senegal

**DOI:** 10.1002/hec.3791

**Published:** 2018-07-05

**Authors:** Seiro Ito, Aurélia Lépine, Carole Treibich

**Affiliations:** ^1^ Institute of Developing Economies Chiba Japan; ^2^ London School of Hygiene and Tropical Medicine London UK; ^3^ University Grenoble Alpes, CNRS, INRA Institute of Engineering Univ. Grenoble Alpes GAEL France

**Keywords:** HIV/AIDS, matching, Senegal, sex work, sexually transmitted infections

## Abstract

Senegal is the only African country where sex work is legal and regulated by a health policy. Senegalese female sex workers (FSWs) are required to register with a health facility and to attend monthly routine health checks aimed at testing and treating sexually transmitted infections (STIs). Compliance to those routine visits is recorded on a registration card that must be carried by FSWs in order to avoid sanctions in case of police arrests. Although this policy was first introduced in 1969 to limit the spread of STIs, there is no evidence so far of its impact on FSWs' health and well‐being. The paper aims to fill this gap by exploiting a unique data set of registered and unregistered Senegalese FSWs. Using propensity score matching, we find that registration has a positive effect on FSWs' health. However, we find that registration reduces FSWs' subjective well‐being. This finding is explained by the fact that registered FSWs are found to engage in more sex acts, in riskier sex acts, have less social support from their peers, and are more likely to experience violence from clients and police officers. We prove that those results are robust to the violation of the conditional independence assumption, to misspecification of the propensity score model, and that covariate balance is achieved. The results suggest that more efforts should be deployed to reduce the stigma associated with registration and to address the poor well‐being of FSWs, which is counterproductive to HIV prevention efforts.

## 1 INTRODUCTION

The legal status of sex work varies widely across countries worldwide, but sex work is illegal in most countries and where legal, soliciting, pimping, or running brothels often remain illegal. The reason for prohibiting sex work lies in moral concerns and on the idea that legalization could increase the spread of HIV and sexually transmitted infections (STIs) by increasing the number of commercial sex acts. However, because criminalization is associated with greater isolation and stigma toward female sex workers (FSWs) and clients (Weitzer, 2005), some studies highlighted that criminalizing sex work translates into more risk‐taking and leads to greater STI transmission (Cameron, Muz, & Shah, 2016; Cunningham & Shah, 2014; Gertler & Shah, 2011).

Although most countries have acknowledged sex work as a public health concern, only a few have used registration and monitoring of STIs in FSWs as a policy to control the spread of HIV/AIDS in the general population, and Senegal is currently the only African country where sex work is regulated by a public health policy. Indeed, given that STIs increase both the risk of new infections among HIV‐negative people and the risk of transmission from HIV‐positive people (Galvin & Cohen, 2004), STI treatment is a highly cost‐effective HIV prevention strategy (Gilson et al., 1997), particularly among FSWs (Steen & Dallabetta, 2003). Since 1969, Senegalese FSWs aged more than 21 years old have been compelled to register with a health center and to attend routine health visits in order to be tested and treated for STIs and to receive free condoms (Chersich et al., 2013). An official registration card is issued (called “*carnet sanitaire*”) to keep a record of the visits made to the appointed health center. If FSWs are tested positive for any STI, with the exception of HIV, the card is kept at the health center during the whole course of treatment. Screened HIV‐positive FSWs will be linked into care, and adherence to antiretroviral treatment will be monitored during routine visits, hence limiting the spread of the disease. FSWs who fail to present an up‐to‐date registration card (either because they are not registered, do not comply with routine visits, or are currently being treated for STI) may incur a prison sentence of between 2 and 6 months (cf. Code pénal articles 319/ 325). The registration policy was inherited from the French colonial rule and find its root in the medical and sanitary policies that were elaborated at the federal level until independence in 1960 (Becker & Collignon, 1999). Since its introduction, no significant changes in this policy were implemented, except for minor adjustments in the appearance of the carnet sanitaire.

Despite its legal status, sex work is morally condemned by society members in Senegal, and keeping sex work secret is a central preoccupation of Senegalese FSWs. Becoming a registered FSW increases the probability that the sex work activity will be discovered because first, FSWs need to carry and hide their concealed registration card while at home; second, registered FSWs will meet clients in public places ; third, compulsory visits for FSWs are held on a specific day (Thursday); and finally, personal information of registered FSWs will remain in police records even if they leave sex work. The fear of becoming a social outcast acts as a main barrier to registration and explains that 80% of FSWs in Senegal (Foley & Nguer, 2010) and 57% in the capital city, Dakar, are not registered (APAPS & IRESSEF, 2015). This justifies that FSWs are still a main contributor to the HIV epidemic: with a prevalence of 6.6%, they are up to 9 times more likely to be infected with HIV than the general population (APAPS & IRESSEF, 2015).

Although many studies investigate market‐level effects of legalizing sex work, a few studies provide causal evidence. Among these, only two studies consider a context where sex work is legal and regulated by a public health intervention. The seminal work by Gertler and Shah (2011) consider the changes in police raid probabilities under an existing registration policy in Ecuador. Using survey data of FSWs containing biological markers, they show two conflicting impacts on STI incidence: an increase in the brothel sector and a decrease in the street sector. Quast and Gonzalez (2017) consider a registration policy implemented in Tijuana, Mexico, that closely resembles the Senegalese setting that we consider in this paper. The policy was introduced in 2005 and is shown to have led to a decrease in the incidence rate of trichomoniasis in the general population by 37% between 2005 and 2012. Although this latter paper finds an overall beneficial effect of this public health policy on STI prevalence at the population level, it does not uncover possible complex behavioral responses.

In order to fill this gap in knowledge, this paper aims to evaluate the impact of becoming a registered FSW in Senegal on both health and subjective well‐being of sex workers and to identify pathways of impacts. To do so, we first develop a theoretical framework that models the main effects of registration on health and well‐being and points out the different channels at play. Based on the theoretical model, we show that the effect of registration on health is both positive and negative: registration leads to a greater number of sex acts, which translates to a higher incidence rate of STIs but, at the same time, is associated with greater investment in health capital through the prevention and early treatment of STIs. We also show that registration deteriorates well‐being through increased stigma, which could partially offset the beneficial health effect of registration, assuming that low well‐being leads to increased risk‐taking (Yuen et al., 2016).

We then test the model's predictions empirically by using a unique data set collected from a sample of 630 FSWs in Dakar, stratified by the registration status. Given the voluntary nature of registration, we use propensity score matching in order to construct a balanced sample of registered and non‐registered FSWs. Our empirical results show that the net effect of registration on STI reduction is positive. Empirical analysis also sheds light on several unintended consequences of the policy on well‐being that may attenuate its positive impact on STIs reduction: registered FSWs engage in more and riskier sex acts, are more likely to experience physical violence, and have less social support from their coworkers. We investigate the effect of the violation of the conditional independence assumption (CIA) by simulating the effect of relevant unobserved confounders affecting both the treatment and the outcomes of interest. We show that the existence of such confounders is unlikely to affect the results. Finally, our empirical results are robust to two additional methods to improve the performance of the propensity score matching: namely, the use of a super learner to improve the specification of the propensity score and the use of entropy balancing in order to achieve covariates balance.

To summarize, this paper contributes to the literature on the regulation of sex work, but, unlike previous studies, it adopts a unique angle by investigating the consequences of registration on FSWs themselves in order to provide a full picture of their behavioral response. The paper also contributes to the literature on social stigma by highlighting the negative unintended effects of a public health policy targeting high‐risk groups to limit the spread of STIs and HIV/AIDS.

The remainder of the paper is organized as follows. In Section 2, we model the theoretical framework. Section 3 presents the data and descriptive statistics. Section 4 presents the method to overcome the selection bias associated with the decision to register along with the sensitivity analysis undertaken to test the violation of the CIA. Results and a series of robustness checks are presented in Section 5 and discussed in Section 6. Finally, Section 7 concludes.

## 2 THEORETICAL FRAMEWORK

### 2.1 Setup

Let us consider a country where prostitution is regulated. Sex workers can, in such context, choose between two types of prostitution: either they choose to solicit clients in public places or they choose to remain discreet. If they choose to solicit clients in public places, they will access a larger pool of clients, but to do so, they need to register with authorities to avoid penal sanctions. Therefore, a sex worker chooses both the number of sex acts *a* and her registration status *R* = {0,1} in order to maximize her utility.

The utility of a FSW under registration status *R* depends on her consumption *c* ^*R*^, expected external stigma *δ* *s*, internal stigma *τ*, and expected legal penalty (1 − *R*)*r* *m*. We assume consumption and various utility costs to be additively separable.

(1) $$U=u(c^{R})-\delta^{R}(a^{R})s(A)-\tau (a^{R})-(1-R)r(a^{R})m.$$

Despite the legalization of prostitution by a government, prostitution is often morally condemned by the society. We consider two kinds of moral costs: *external* stigma *s*, when identified as a sex worker by her relatives, friends, or neighbors; and *internal* stigma *τ*, a FSW suffers from damages of her self‐image and self‐esteem when engaging in commercial sex acts. The chance *δ* of being identified as a sex worker is no smaller under registration, $\delta^{1}⩾\delta^{0}$ for a given *a*. This is a direct consequence of multiple elements related to registration that are implemented in Senegal, such as holding a registration card issued only to FSWs, working in public places, and being registered in police files. 1This assumption of differential probability may seem to be conjectural rather than factual. However, based on anecdotes from focus groups discussions in which they stressed a worry of the registration card to be found by relatives, we believe that this assumption is realistic in the Senegalese context. Our data also show that the main reason for not registering is the preference for discreetness, which hints that FSWs believe $\delta^{1}⩾\delta^{0}$.
^,^ 2We also note that predictions of the model are sharper if we do not impose it and use *δ* ^0^ = *δ* ^1^ or *δ* ^0^ > *δ* ^1^ instead, yet we can still derive the results with $\delta^{1}⩾\delta^{0}$.We assume that *δ* ^*R*^ increases with *a* ^*R*^, $\delta^{\prime }(a^{R})⩾0,\forall a^{R}\in R_{+}$.

We assume that external stigma increases with the level of wealth of the family, $s(A)>0,s^{\prime }(A)>0,\forall A\in R_{+}$, as it may damage a family reputation and a wealthier family may feel more strongly against prostitution. Internal stigma is assumed to be nondecreasing in the number of sex acts, $\tau^{\prime }(a)⩾0$ for all *a*. A legal penalty *m* only applies to non‐registered FSWs. Its sanction probability *r*(*a* ^0^) is assumed to be nondecreasing in *a* ^0^, $r^{\prime }(a^{0})⩾0,\forall a^{0}\in R_{+}$. We assume that *u* is increasing and concave in *c* ( $u^{\prime }(c)>0,u^{\prime \prime }(c)⩽0,\forall c\in R_{++}$). 3For completeness, we note that we assume *s*(0) > 0, that is, that even the poorest FSW feels external stigma if being identified as a sex worker. We further assume that *δ* and *r* follow symmetric, bell‐shaped density functions, which implies that the density is increasing for values below mean and decreasing for values above mean *μ*_*a*_, or $\delta^{R^{\prime \prime }}(a)⩾0$ for $a⩽\mu_{a}$, $\delta^{R^{\prime \prime }}(a)<0$ for otherwise, and $r^{\prime \prime }(a)⩾0$ for $a⩽\mu_{a}$, *r* *′* *′*(*a*) < 0 otherwise.


Given that the non‐registered FSWs cannot work in all venues and are limited to a subset of the market, 4This is confirmed in our database, in which a larger share of non‐registered FSWs operates at home. we conjecture that the market size is bigger for the registered FSWs. So the (inverse) demand is larger for registered FSWs, that is, $w^{0}(a)⩽w^{1}(a)$. 5In Section 3, we found that price distributions for registered and non‐registered FSWs overlap and their means are not statistically significantly different from each other. At the same time, the number of acts is larger for the registered FSWs, and their mean difference is statistically significant. The registered FSWs derive larger incomes from sex acts than the non‐registered FSWs. The strict inequality holds when a bigger registered market has clients who have higher willingness to pay for all sex act levels *a*.In addition, we assume that such a difference in market size is bounded from below in terms of a density ratio $\frac{\delta^{1^{\prime }}}{\delta^{0^{\prime }}}$. We combine these two market size related assumptions as Assumption 2.1.

Assumption 2.1 For all $a\in R_{+}$:

$$\frac{w^{1^{\prime }}(a)a+w^{1}(a)}{w^{0^{\prime }}(a)a+w^{0}(a)}⩾\frac{\delta^{1^{\prime }}(a)}{\delta^{0^{\prime }}(a)}.$$

Intuitively, the inequality in Assumption 2.1 states that the marginal gain in income is greater than the marginal loss in terms of chances of being identified as a sex worker, as one switches from unregisterd to registered. This assumption always holds for a bell‐shaped density with equal dispersions at $a⩽\mu_{a^{1}}$. Note also that it is independent of *A*, so any FSW will choose to supply more sex acts had they registered. This, of course, does not mean that all FSWs will be better off by registering and by supplying a larger number of sex acts.

### 2.2 The FSW's problem

A FSW under registration status *R* solves the following maximization program:

(p1) $$\begin{array}{ccc} \max_{\left\{a^{R}\right\}} & \;\;\;u(c^{R})-\delta^{R}(a^{R})s(A)-\tau (a^{R})-(1-R)r(a^{R})m & \\ s.t. & \;\;\;w^{R}(a^{R})a^{R}+A=c^{R}. \end{array}$$

The first order condition (FOC) is

(2) $$F\equiv u^{\prime }\cdot (w^{R^{\prime }}a^{R}+w^{R})-\delta^{R^{\prime }}(a^{R})s(A)-\tau^{\prime }(a^{R})-(1-R)r^{\prime }(a^{R})m=0.$$

As the marginal income is larger for the registered $w^{1^{\prime }}(a)a+w^{1}(a)⩾w^{0^{\prime }}(a)a+w^{0}(a)$, for a given *A*, Equation 2 immediately gives that $a^{1}⩾a^{0}$.

**Figure 1:**
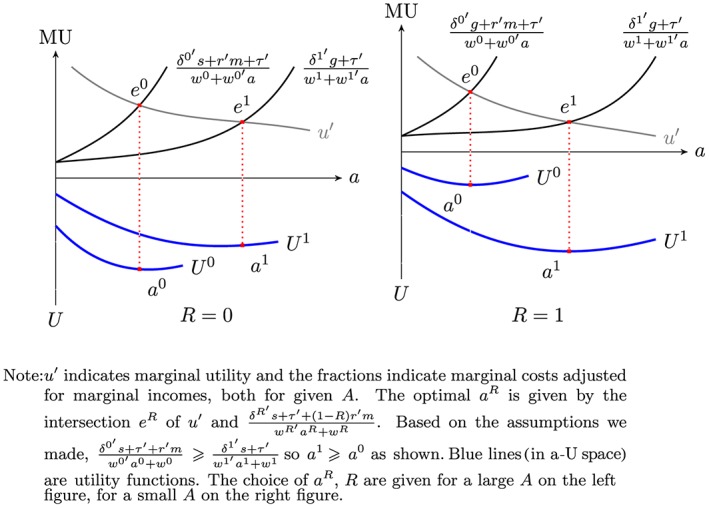
A large A case (left) and a small A case (right) [Colour figure can be viewed at http://wileyonlinelibrary.com]

In Figure 1, the optimal *a* ^*R*^ is given by the intersection *e* ^*R*^ of *u* *′* and $\frac{\delta^{R^{\prime }}s+\tau^{\prime }+(1-R)r^{\prime }m}{w^{R^{\prime }}a^{R}+w^{R}}$.

To make sure that the solution is a maximizer, we assume the following (for more details on the concavity of the maximization problem, see Appendix A.1.4):

Assumption 2.2 Internal stigma *τ* is an increasing, convex function of *a*, such that it dominates the decrease in density $\delta^{\prime \prime }$ and *r* *′*(*a*) for any level of *A* and *m* for a large *a*:

$$\delta^{R^{\prime \prime }}(a)s(A)+\tau^{\prime \prime }(a)+(1-R)r^{\prime \prime }(a)m⩾0.$$

For a small *a* under bell‐shaped density for $\delta^{\prime \prime }$ and *r* *′*, this is always satisfied.

A FSW will register if the value function under registration *V* ^1^ is larger than the one under illegality *V* ^0^. Because the FOC depends on *A* through marginal utility and marginal expected external stigma, the decision to register also depends on *A*. A FSW decides to register if

(3) $$\begin{array}{ccc} V^{0}(A) & =u\left\{w\left(a^{0}\right)a^{0}+A\right\}-\delta^{0}\left(a^{0}\right)s(A)-\tau \left(a^{0}\right)-r\left(a^{0}\right)m & \\ & <u\left\{w^{1}\left(a^{1}\right)a^{1}+A\right\}-\delta^{1}\left(a^{1}\right)s(A)-\tau \left(a^{1}\right)=V^{1}(A). \end{array}$$

By using the envelope theorem, one can show that *V* ^*R*^ is increasing in *A* and the slope is greater for *V* ^0^ than *V* ^1^. So the inequality (3) is likely to hold for a small *A*, which we summarize as Assumption 2.3.

Assumption 2.3 Equation 3 holds for a small enough *A*.

**Figure 2:**
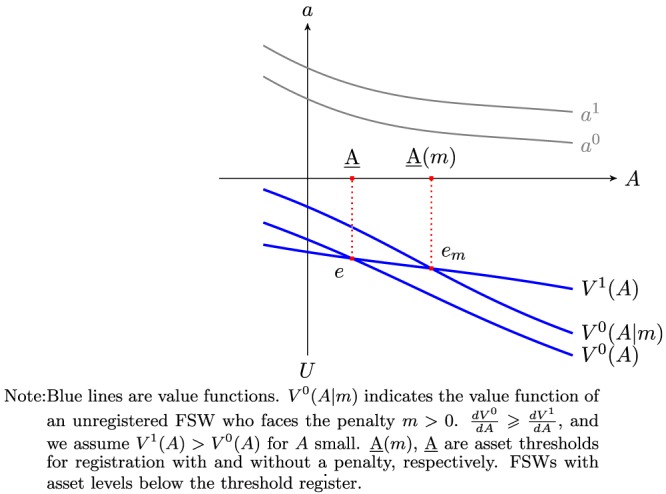
Registration decision over A. FSWs: female sex workers [Colour figure can be viewed at http://wileyonlinelibrary.com]

Given that external stigma depends positively on household wealth, one can show that the decision to register *R* switches from 0 to 1 as *A* becomes smaller. That is, there exists *A*_ such that *V* ^1^(*A*) > *V* ^0^(*A*) for *A* ≤ *A*_ as shown in Figure 2. We note that a FSW with a smaller *A* provides more sex acts hence finds more benefits in registration:

$$\frac{da^{R}}{dA}=-\frac{F_{A}}{F_{a}}=-\frac{u^{\prime \prime }\cdot (w^{R^{\prime }}a^{R}+w^{R})-\delta^{R^{\prime }}(a^{R})s^{\prime }(A)}{\text{SOC}}.$$

The denominator is the SOC $=u^{\prime \prime }\cdot {\left(w^{R^{\prime }}a^{R}+w^{R}\right)}^{2}+u^{\prime }\cdot (2w^{R^{\prime }}+w^{R^{\prime \prime }}a^{R})-\{\delta^{R^{\prime \prime }}s+\tau^{\prime \prime }(a)+(1-R)r^{\prime \prime }(a)m\}$ that holds strictly under the assumptions we have made (SOC =*F*_*a*_ < 0 ). This is depicted as negatively sloped value functions in Figure 2. A penalty *m* increases the marginal cost of supplying *a*, so it decreases the number of sex acts *a* ^0^ by the non‐registered FSWs. In Figure 2, it shifts down the value function of non‐registration, changing the intersection to *b*_*m*_ and thus the associated threshold asset to a larger level denoted as *A*_(*m*) with *A*_*′*(*m*) > 0, *A*_(*m*) > *A*_(0) for $m\in R_{++}$. A larger penalty induces FSWs with a larger *A* to register.

### 2.3 Results

All the results obtained are summarized in the following propositions and corollaries.

Proposition 2.1 There exists *A*_ such that $V^{1}(A)⩾V^{0}(A)$ for *A* ≤ *A*_: For a small enough level of wealth, a FSW decides to register.

Proposition 2.2 $a^{1}⩾a^{0}$: For a given level of asset *A*, a registered FSW performs more sex acts than an unregistered FSW.

Proposition 2.3 *a* ^*R*^ decreases with the level of asset *A*.

Proposition 2.4 *A*_ < *A*_(*m*): A legal penalty of nonregistration induces FSWs with a larger *A* to register.

Corollary 2.1 *w* ^1^(*a* ^1^)*a* ^1^ ≥ *w* ^0^(*a* ^0^)*a* ^0^: A registered FSW earns more.

Corollary 2.2 *δ* ^1^(*a* ^1^)*s*(*A*) ≥ *δ* ^0^(*a* ^0^)*s*(*A*) and *τ*(*a* ^1^) ≥ *τ*(*a* ^0^): A registered FSW suffers from greater external and internal stigma.

In Appendix A.1.3, we introduce health risks under an assumption that registration reduces the expected health costs through consultation and treatment. We show that all the propositions continue to hold along with additional results on health:

Proposition 2.5 The greater the probability *β* ^*R*^ of being cured, the greater the sex act supply *a* ^*R*^. The access to quality health services increases the number of sex acts.

Proposition 2.6 The greater the damage *I*, the smaller the sex act supply *a* ^*R*^. The severity of a disease reduces the number of sex acts.

We remark the following:

Remark 2.1 If the relative risk of being infected for registered FSWs compared with non‐registered FSWs is lower (greater) than the relative probability of being treated, that is, $\frac{\pi (a^{1})}{\pi (a^{0})}<(>)\frac{\left(1-\beta^{0}\right)}{\left(1-\beta^{1}\right)}$, registration ensures a lower (greater) expected physical health damage.

## 3 DATA AND DESCRIPTIVE STATISTICS

Data were collected from 320 registered and 310 unregistered FSWs over 21 years of age and living in Dakar suburbs in June and July 2015, which represents 15% of the estimated total FSWs in the region of Dakar (APAPS, 2011–2012). Registered FSWs were recruited by the midwife in charge of their medical follow‐up. All active registered FSWs from four (Pikine, Rufisque, Mbao, and Sebikotane) out of the five STI health centers located in Dakar were contacted to participate in our study. Unregistered FSWs were recruited by FSWs' group leaders. FSWs were asked to come to the health center and were interviewed at the health facility in private dedicated rooms. Survey participants receive a CFAF 3,000 show up fee that aimed to cover transport cost and the time spent at the health facility.

We measure physical health by the probability of being sick over the last 4 weeks and by the presence of lower abdominal pain in the last month because lower abdominal pain, unlike vaginal discharge (Desai et al., 2003), has been shown to be strongly correlated with pelvic inflammatory disease (Bentsi et al., 1985). In the sample, 42% had an illness episode over the last 4 weeks, and 12% suffered from lower abdominal pain. Subjective well‐being is measured by happiness level, life satisfaction in general, and self‐esteem. Data highlight very low level of well‐being in our sample: 30% of FSWs declare that they are not happy, 25% that they are not at all satisfied with their lives, and 15% strongly disagree with the statement “Overall, I am satisfied with myself.”

Furthermore, information on their use of prevention services, their sex work environment, and their social networks was also collected. Table 1 presents the descriptive statistics of registered and unregistered FSWs. On average, sampled FSWs are 36 years old and have low level of education. Most FSWs are divorced, and among these, only 7% receive any spousal maintenance from their ex‐partner, which is consistent with the fact that 92% of FSWs report to have entered the sex work market because of financial reasons. Regarding household composition, they live in households of six persons on average: 34% live with their parents, 62% with their children, and 48% with their brother, and they have two children. Regarding sex work activities, on average, FSWs have been in the sex work business for 8 years. FSWs report a monthly earning of USD 229 (CFAF 133,492), have monthly household expenditure of USD 607 (CFAF 353,881), and have monthly per capita expenditure of USD 165 (CFAF 96,520), which is 2.2 times higher than the level of per capita expenditure in Dakar reported in the national statistics (CFAF 43,260; ANSD, 2013). In the sample, the demand for HIV and STI prevention is high given that 74% of FSWs are affiliated to a STI center and 57% have visited the STI center less than a month ago. Self‐reported condom use is also high, as 98% report to have used a condom with the last client, however when elicited indirectly via a list experiment, condom use rate dropped to 78% (Treibich & Lépine, 2016). In general, FSWs tend to self‐report taking low risks in sexual behaviors: Only 2% report to have engaged in anal sex, and 6% report to have had sex with more than one client at a time.

**Table 1:** Descriptive statistics

	All FSWs		Unregistered FSWs		Registered FSWs		
Variables	*N*	Mean	*N*	Mean	*N*	Mean	*p* value
*Sociodemographic characteristics*							
Age (in years)	630	36.42	310	36.01	320	36.81	0.257
Divorced (%)	630	0.708	310	0.668	320	0.747	0.029
No education (%)	629	0.278	310	0.226	319	0.329	0.004
Has at least one child (%)	630	0.888	310	0.897	320	0.881	0.536
Father or mother lives in Dakar (%)	630	0.548	310	0.626	320	0.472	0.000
Preference towards the future (%)	630	0.210	310	0.181	320	0.238	0.080
Altruism (USD)	630	0.47	310	0.37	320	0.58	0.000
Risk aversion (CRRA based on Gneezy and Potters game)	630	0.754	310	0.759	320	0.749	0.859
Beauty (score out of 10)	630	5.78	310	5.85	320	5.71	0.308
Entered the sex business alone (%)	630	0.532	310	0.577	320	0.488	0.024
Fatality (%) ^a^	628	0.635	309	0.702	319	0.571	0.001
Own house (%)	630	0.200	310	0.268	320	0.134	0.000
*Final outcomes*							
Physical health							
Has been sick or injured in the past 4 weeks	630	0.419	310	0.461	320	0.378	0.035
Had lower abdominal pain in the past month	629	0.116	310	0.145	319	0.088	0.025
Well‐being							
Is not happy	630	0.303	310	0.258	320	0.347	0.015
Is not at all satisfied with her life in general	629	0.245	310	0.210	319	0.279	0.043
Strongly disagree with							
“Overall I am satisfied with myself”	629	0.146	310	0.106	319	0.185	0.005
*Intermediate outcomes*							
Prevention							
Received free condoms	621	0.680	303	0.472	318	0.877	0.000
Is affiliated to an STI center	627	0.740	308	0.542	319	0.931	0.000
Went to an STI center in the last month	630	0.567	310	0.274	320	0.850	0.000
Had an HIV screening in the past year	630	0.810	310	0.674	320	0.941	0.000
Sought care for last STI	267	0.775	112	0.768	155	0.781	0.806
Sought care for last illness	630	0.721	310	0.710	320	0.731	0.547
Unhealthy behaviors							
Cigarette expenses in the last 7 days	627	1,152	310	895	317	1,403	0.096
Alcohol expenses in the last 7 days	627	984	309	347	318	1,602	0.003
Sex work environment							
Number of clients per week	627	6.514	310	5.145	317	7.852	0.000
Usually attracts clients in bars or nightclubs	630	0.421	310	0.245	320	0.591	0.000
Last client was an occasional client	624	0.442	307	0.358	317	0.524	0.000
Had alcohol before last sex act	619	0.076	305	0.039	314	0.111	0.001
Last client consumed alcohol	617	0.152	306	0.085	311	0.219	0.000
Last sex was with more than one client	583	0.062	297	0.037	286	0.087	0.012
Used a condom during last sex act	562	0.977	296	0.973	266	0.985	0.327
Anal intercourse during last sex act	624	0.022	307	0.013	317	0.032	0.119
Fellatio during last sex act	624	0.064	307	0.059	317	0.069	0.584
Is not satisfied at all with sex work	627	0.418	310	0.342	317	0.492	0.000
In the past year:							
Suffered from violence by an occasional client ^b^	364	0.291	177	0.243	187	0.337	0.049
Suffered from police violence ^b^	553	0.061	310	0.039	243	0.091	0.012
Fear of police							
In the past year:							
Reported violence incident to the police ^b^	74	0.257	32	0.188	42	0.310	0.240
Earnings and savings							
Monthly earnings from sex work (FCFA)	628	133,492	310	100,461	318	165,692	0.000
Savings in the past month (FCFA)	624	15,964	308	3,482	316	28,131	0.000
Leaving sex work							
Is sure that she will no longer							
be a FSW in 3 years ^c^	563	0.355	278	0.371	285	0.340	0.456
Social network							
Rivalry ^d^	630	139	310	87	320	188	0.000
Altruism towards other FSWs	630	137	310	125	320	148	0.174
Satisfaction in friendship domain ^e^	612	2.466	304	2.559	308	2.373	0.011
Has at least one FSW to go for reassurance	603	0.474	306	0.533	297	0.414	0.004
Has at least one FSW to borrow money from	603	0.393	306	0.438	297	0.347	0.022
Stigma							
Family knows about her sex work activity	620	0.281	306	0.235	314	0.325	0.013
Life satisfaction with family ^e^	629	2.571	309	2.654	320	2.491	0.024

*Note*. *N* stands for the number of observations. FSWs: female sex workers; STI: sexually transmitted infection. Fatality  =  0 if strongly disagree with “if someone is meant to have a disease he will get it.” FSWs who registered less than a year before the interview were excluded. FSWs who did not understand the proposed scale were excluded. Rivalry is measured as the difference in the amount given to an NGO that takes care of street children and the amount given to another FSW in two dictator games. Life satisfaction is measured on a scale from 1 to 4, with a higher number denoting higher life satisfaction.

## 4 METHOD

### 4.1 Propensity score matching

In order to evaluate the impact of the registration policy, one would ideally need to compare the outcome *Y*_1_ for a registered FSW (*R* = 1) with the outcome *Y*_0_ that we would observe if this FSW was not registered (*R* = 0). Unfortunately, because FSWs are either registered or unregistered, these two sets of outcomes are never observed for a same FSW. If the registration status was randomly assigned, a simple difference in outcome means between legal and illegal FSWs would provide accurate estimates of the impact of the policy. However, given that FSWs register to the policy on a voluntary basis, registered FSWs may not closely resemble unregistered ones as shown in Table 1. In order to control for the selection bias due to observables, we use propensity score matching.

Following the notation of Blundell and Costa Dias (2000), we want to estimate the effect of being a registered FSW given by

(4) $$\alpha =E(Y_{1}-Y_{0}|X,R=1).$$

This is the expected difference in the outcomes between the treated and the control after accounting for the observable characteristics (*X*).

The aim of matching is to pair every registered FSW with similar FSWs from the control group based on their observable characteristics (*X*). Our analysis relies on the CIA that the outcomes of the non‐treated FSWs (*Y*_0_) and treated FSWs (*Y*_1_) are independent of the registration status *R* once one controls for (*X*).

(5) $$(Y_{0},Y_{1})\;⊥\;R|X.$$

Given the high dimension of *X*, a more feasible option is to match on a function of *X*. It has been shown that the probability to register *P*(*X*), or the propensity score, can serve as such a function under the overlap assumption, which states that FSWs who are similar along the selected set of observable characteristics (*X*) must have a strictly positive probability to be either treated or untreated:

(6) 0<Pr(R=1|X)<1.

Hence, we matched on the propensity score to create a balanced sample. CIA and the overlap assumption are combined as the strong ignorability assumption (Rosenbaum & Rubin, 1983):

(7) $$(Y_{0},Y_{1})\;⊥\;R|P(X).$$

Once the closest matches among the controls have been found, we use the Gaussian Kernel matching estimator that matches all treated units with a weighted average of all controls with weights that are inversely proportional to the distance between the propensity scores of treated and controls. Valid standard errors were obtained by bootstrapping.

(8) $$\alpha_{t}=\frac{1}{N_{T}}\sum_{i\in T}\left\{Y_{i}^{T}-\frac{\sum_{j\in C}Y_{j}^{C}G\left(\frac{p_{j}-p_{i}}{h_{n}}\right)}{\sum_{k\in C}G\left(\frac{p_{k}-p_{i}}{h_{n}}\right)}\right\},$$

where *N*_*T*_ is the number of units in the treated group *i* and *N*_*C*_ in the control group *j*, *p* is the propensity score, *G*(.) is a kernel function, and *h*_*n*_ is a bandwidth parameter.

Let *S* ^⋆^ be the space of *X* that is simultaneously observed among registered and non‐registered FSWs (common support of *X*). The expected average effect of the registration policy among treated participants (ATT) for whom we can find a comparable non‐treated match is given by

(9) $$\frac{\int_{S^{⋆}}E(Y_{1}-Y_{0}|P(X),R=1)dF(P(X)|R=1)}{\int_{S^{⋆}}dF(P(X)|R=1)}.$$

### 4.2 Sensitivity analysis

Our main analysis is based on the CIA, which assumes that there is no unobservable characteristic that explains both the decision to register and the outcomes of interest. Ichino, Mealli, and Nannicinni (2008) propose a simulated sensitivity analysis to test whether the results obtained with the propensity score matching are robust to specific failures of the CIA. The idea is to assume that the selection on observables assumption is not satisfied given the observables considered (Equation 10) but could be if one could observe an additional binary variable *U* (Equation 11).

(10) $$(Y_{0},Y_{1})\;⊥\;/\;R|X.$$

(11) $$(Y_{0},Y_{1})\;⊥\;R|X,U.$$

We assume that the unobserved variable may impact both the treatment and the outcome. To describe the distribution of the hypothetical confounding variable *U* completely, we can define four probabilities *p*_*ij*_ where the treatment status *R* and the outcome *Y* are observed in the data:

(12) $$p_{ij}=Pr(U=1|R=i,Y=j).$$

Ichino et al. (2008) define the selection effect Λ as the effect of *U* on the relative probability to be assigned to the treatment and the outcome effect Γ as the effect of *U* on the relative probability to have a positive outcome in the absence of treatment.

(13) $$\Lambda =\frac{\frac{P(R=1|U=1,X)}{P(R=0|U=1,X)}}{\frac{P(R=1|U=0,X)}{P(R=0|U=0,X)}}.$$

(14) $$\Gamma =\frac{\frac{P(Y=1|R=0,U=1,X)}{P(Y=0|R=0,U=1,X)}}{\frac{P(Y=1|R=0,U=0,X)}{P(Y=0|R=0,U=0,X)}}.$$

Put differently, an outcome effect of Γ >1(<1) means that the unobserved *U* positively (negatively) affects the outcome variable. Similarly, a selection effect of Λ >1(<1) means that the unobserved *U* increases (decreases) the probability to register.

In order to study the robustness of the results obtained with propensity score matching to the violation of the CIA, Ichino et al. (2008) propose two alternatives. A first approach relies on the assumption that the unobserved variable *U* should have a similar selection and outcome effects than the ones of covariates. To implement this test, we fix *p*_*ij*_ = *P*(*U* = 1|*R* = *i*,*Y* = *j*) according to their values for the set of covariates used in the propensity score model. A second approach is to search for the existence of parameters *p*_*ij*_ such that if *U* were observed, the estimated ATT would be driven to zero. To do so, we simulate all possible distributions of *U* 6We fixed *p*_11_ = *p*_10_ and then varied the values of *p*_11_, *p*_01_, *p*_00_ from 0.1 to 0.9 (Ichino et al., 2008). and test the plausibility of the configurations of parameters killing the ATT. If the distribution of *U* killing the ATT is considered unlikely, this exercise would support the robustness of the estimates derived under CIA.

## 5 RESULTS

In this section, we first model the decision to register with authorities and estimate the propensity score. In the second step, we present the average treatment effects on the treated (ATT) using Kernel matching. We then implement the sensitivity analysis to study the consequences of the violation of the CIA on the main findings. Finally, we investigate the robustness of the results by using multivariate reweighting method to produce balanced samples and by further improving the specification of the propensity score by using machine learning.

### 5.1 Determinants of registration

#### 5.1.1 Determinants of registration

Table 2 presents the determinants of registration and includes observable characteristics that may affect the decision to register with authorities but that we consider to be exogenous to outcomes. To choose the most relevant set of covariates, we use predetermined variables and elicited preferences that we assume to influence the decision to register.

**Table 2:** Determinants of registration

Variables	Coefficient	Robust *SE*
Age	0.000	0.002
Divorced	0.076*	0.044
No education	0.059	0.043
Has at least one child	−0.085	0.062
Father or mother live in Dakar	−0.118***	0.041
Preference towards the future	0.071	0.047
Altruism	0.209***	0.042
Risk aversion	0.010	0.027
Beauty	−0.049	0.048
Enters the sex business alone	−0.085**	0.038
Fatality	−0.096**	0.041
Own house	−0.161***	0.049
Observations	627	
*R* ^2^	0.119	
VIF (max)/(mean)	1.28/1.09	

*Note*. VIF stands for variance inflation factors and is used to test multicollinearity of independent variables. Binary variable which equals 1 if the women gave more than 40% of the money received in a dictator game to a street children association.

* *p*< 0.1.

** *p*< 0.05.

*** *p*< 0.01.

Among the non‐registered FSWs, the main reason against registration was the willingness to remain discreet (44%); followed by the fact that their sex work activity was occasional (18%), the poor knowledge of the legal system (10%), or procrastination (4%). Hence, a key determinant of registration is associated with the fear of being a social outcast. To account for this, we include whether the FSW's parents live in Dakar as this would increase the risk of being discovered by relatives. In addition, we include whether the FSW was introduced to sex work by another FSW assuming that those FSWs would be more likely to be aware of the registration policy than those who entered sex work alone. It is assumed that FSWs who have other opportunities outside sex work may decide to do this activity only occasionally. We thus control for a set of variables that may be correlated with the FSW's opportunity cost such as educational level to account for outside work opportunities, but also age and beauty to account for the fact that younger or more attractive FSWs will be more willing to meet clients in bars and nightclubs and hence will have greater benefits in becoming registered FSWs. We also control for whether FSWs are divorced and whether they have children as this may be correlated with their economic vulnerability.

Among registered FSWs, health concerns (62%) and police threats (36%) were the main reasons for registering. In order to account for these factors, we control for the following intrinsic attributes: risk aversion in the financial domain (elicited via the Gneezy and Potters game with real payments), 7Although risk aversion in money may not directly be correlated with risk aversion in health, we expect that it will predict the decision of register through financial losses resulting from the fine.self‐reported preference for the present, altruism (elicited via a dictator game with real payment where the recipient was a street child), and self‐reported fatalism. Moreover, we control for whether the FSW's household owns the house she lives in. Contrary to the observed assets, which are a mix between the FSW wealth prior to her entry in the sex industry and the earnings she has accumulated, we assume that house ownership was more likely to have occured before entering sex work.

The selected set of covariates appears to significantly explain the registration status. Table 2 shows that 12% of the variance in registration status is explained by the model. In particular, and as expected from the theoretical predictions, FSWs who face more economic and social vulnerability are more likely to register.

**Figure 3:**
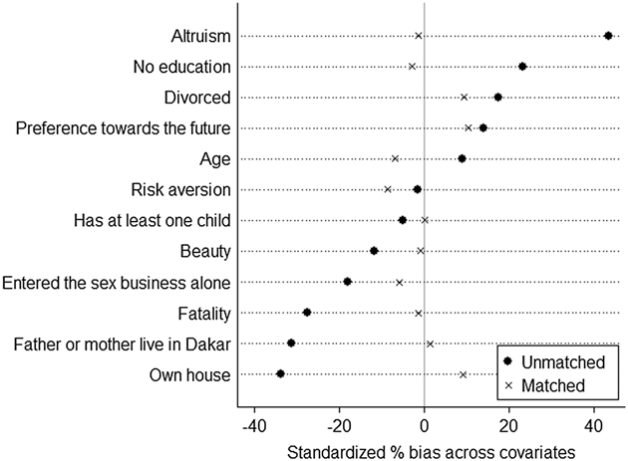
Balance of covariates [Colour figure can be viewed at http://wileyonlinelibrary.com]

In order to test if the matching procedure is able to reduce the mean differences in observed characteristics between registered and non‐registered FSWs, we present the standardized percentage differences in covariates before and after matching. As shown in Figure 3, those differences are considerably reduced, going from up to 43% before matching to less than 10% for all covariates.

#### 5.1.2 Overlap

**Figure 4:**
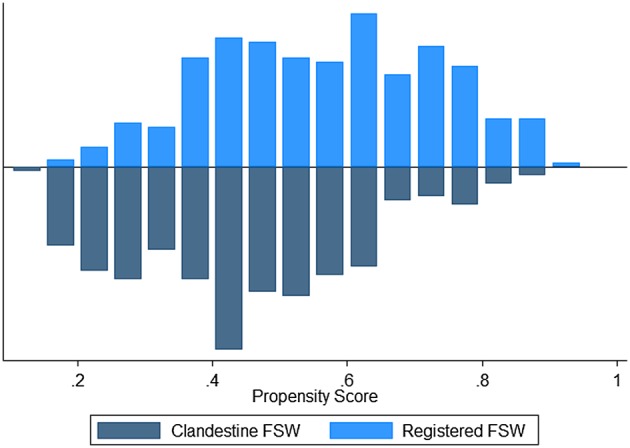
Common support. FSW: female sex worker [Colour figure can be viewed at http://wileyonlinelibrary.com]

Figure 4 brings evidence that the overlap condition is fulfilled by representing the densities of the distribution of the estimated propensity score for registered and non‐registered FSWs. In fact, less than 1% of observations in our sample are off support.

### 5.2 Effects of the policy

Tables 3 and 4 report the average treatment effect on the treated (ATT).

**Table 3:** Registration policy impacts—Final outcomes

						Mean of
	Number of	Number of				matched
Outcomes	treated	controls	ATT	*SE*	Sign.	controls
Physical health						
Has been sick or injured in the past 4 weeks	318	308	−0.109	0.043	**	0.483
Had lower abdominal pain in the past month	317	308	−0.055	0.031	*	0.143
Well‐being						
Is not happy	318	308	0.077	0.041	*	0.266
Is not satisfied at all in general	317	308	0.074	0.037	**	0.207
Strongly disagree with						
“Overall I am satisfied with myself”	318	308	0.090	0.028	***	0.095

*Note*. ATT stands for average treatment effect on the treated. Results of the Gaussian Kernel matching on the common support with replacement and 1,000 replications are presented here.

* *p*< 0.1.

** *p*< 0.05.

*** *p*< 0.01.

**Table 4:** Registration policy impacts—Intermediate outcomes

						Mean of
	Number of	Number of				matched
Outcomes	treated	controls	ATT	*SE*	Sign.	controls
Prevention						
Received free condoms	316	301	0.369	0.039	***	0.508
Is affiliated to an STD center	317	306	0.364	0.039	***	0.566
Went to a health center in the last month	318	308	0.560	0.036	***	0.289
Had an HIV screening in the past year	318	308	0.237	0.033	***	0.707
Sought care last STI	154	110	0.025	0.075	NS	0.760
Sought care last illness	301	305	0.055	0.041	NS	0.716
Unhealthy behaviors						
Cigarette expenses in the last 7 days	315	308	560	325	*	850
Alcohol expenses in the last 7 days	316	307	1300	413	***	289
Sex work environment						
Number of clients per week	315	308	2.665	0.517	***	5.215
Attracts usually clients in bars or nightclubs	318	308	0.338	0.040	***	0.254
Last client was an occasional client	315	305	0.153	0.043	***	0.368
Had alcohol before last sex act	312	303	0.062	0.024	**	0.047
Last client consumed alcohol	310	304	0.125	0.033	***	0.091
Multiple clients relationship during last sex act	285	295	0.051	0.022	**	0.036
Used a condom during last sex act	265	294	0.016	0.016	NS	0.968
Anal intercourse during last sex act	315	305	0.019	0.013	NS	0.013
Fellatio during last sex act	315	305	0.015	0.021	NS	0.054
Is not satisfied at all with sex work	315	308	0.102	0.044	**	0.393
In the past year:						
Suffered from violence of an occasional client	186	168	0.101	0.052	*	0.238
Suffered from police violence	242	308	0.047	0.024	*	0.044
Fear police						
In the past year:						
If suffered from client violence						
went to report it to the police	42	26	0.192	0.094	**	0.117
Earnings and savings						
Monthly earnings from sex work (FCFA)	316	308	62,793	114,77	***	103,552
Savings in the past month (FCFA)	319	320	24,332	5,923	***	3,534
Leaving sex work						
Is sure that she will no longer						
be a FSW in 3 years	283	276	−0.066	0.044	NS	0.405
Social network						
Rivalry	318	308	54	24	**	132
Altruism towards other sex worker	323	320	2	19	NS	147
Life satisfaction with friends	306	302	−0.255	0.080	***	2.624
Has at least one FSW to go to be reassured	296	304	−0.146	0.047	***	0.558
Has at least one FSW to borrow money to	296	304	−0.122	0.046	***	0.467
Stigma						
Family knows about her sex work activity	312	304	0.080	0.041	**	0.244
Life satisfaction with family	318	307	−0.219	0.075	***	2.710

*Note*. NS stands for “not significant.” ATT stands for average treatment effect on the treated. Results of the Gaussian Kernel matching on the common support with replacement and 1,000 replications are presented here. FSW: female sex worker; STI: sexually transmitted infection.

* *p*< 0.1.

** *p*< 0.05.

*** *p*< 0.01.

#### 5.2.1 Effect on self‐reported health

Overall, the net effect of registration on health is positive. We find that registered FSWs are 11 percentage points (23%) less likely to have been sick or injured in the past 4 weeks. They also are 6 percentage points (38%) less likely to have suffered from lower abdominal pain in the past 30 days.

#### 5.2.2 Effect on well‐being

Overall, the net effect of registration on well‐being is negative. Registered FSWs are 8 and 7 percentage points (29% and 36%) more likely to declare that they are unhappy and unsatisfied with their life in general, respectively. Finally, registered FSWs appear to have a lower self‐esteem because they are 9 percentage points (95%) more likely to strongly disagree with the statement “Overall I am satisfied with myself.” Despite assuming that registration has a deterring effect on self‐esteem, we do not find that registration leads to a greater expectation of being in sex work in 3 years of time.

#### 5.2.3 Effects on earning

Registration leads to a change in the place of sex work. Registered FSWs are 34 percentage points (133%) more likely to work in bars or nightclubs, and this translates into a greater activity: Registered FSWs have on average 2.7 (51%) more clients a week and greater earnings. We find that registered FSWs earn CFAF 62,793 (USD 107) or 61% more than unregistered FSWs. This difference in earnings is only explained by a greater intensity of their sex work activity rather than by a higher price charged per sex act. Indeed, there is no difference in the price charged by registered and non‐registered FSWs during the last sex act with both regular clients and occasional ones. In addition, the increase in earnings allows to increase savings level, we find that registered FSWs save 24,332 (USD 41) more than unregistered FSWs.

#### 5.2.4 Unintended effects

##### Change in risk taking

Based on information collected on the circumstances of the last paid sexual intercourse, it appears that the differences in sex work environment also translate into riskier sexual behaviors. More precisely, legal FSWs are 15 percentage points (42%) more likely to have had an occasional client as the last client, 6 percentage points (132%) more likely to have consumed alcohol prior the sex act, and 13 percentage points (137%) more likely to have had sex with an intoxicated client, which may explain that registration leads to a higher likelihood of experiencing violence from a client by 10 points (42%). No difference in condom use or anal sex is detected between registered FSWs and unregistered FSWs, perhaps because of the little variability in those variables (98% of FSWs declare having used a condom and not having performed anal sex during the last sex act). However, registered FSWs were 5 percentage points (141%) more likely to have had a sex act with multiple clients during last sex act. The results also show that because of the accrued contact with the police, registered FSWs are also more likely to have experienced violence from a police officer in the past 12 months by 5 points (125%). However, they are also more likely to report clients' violence to the police.

##### Social support

Greater competition among registered FSWs translates into lower psychological support from peers. We find that registered FSWs are 15 and 12 percentage points (26% in both cases) less likely to report to know a FSW who will reassure her when she needs it and from whom she can borrow money, respectively. This rivalry between FSWs is also detected when comparing the difference in the amount that registered FSWs agree to give to street children and to a peer FSW in dictator games: Registered FSWs give 54 FCFA more (41%) to street children than to a peer in a dictator game than the difference given by unregistered FSWs. This unintended effect may provide another explanation for the negative effect of registration on well‐being.

### 5.3 Sensitivity analysis

In order to further investigate the extent of biases due to confounding factors, we conduct the sensitivity analysis suggested by Ichino et al. (2008) to test whether the results obtained with the propensity score matching are robust to the violation of the CIA.

More precisely, we implement the sensitivity exercise for different outcomes of interest, namely, STI symptoms and low self‐esteem. One may think of several unobserved variables that are likely to simultaneously influence their decision to participate in the program and their physical health or well‐being. In particular, one may argue that intra‐family sexual abuse during childhood may, on the one hand, explain the family breakdown and therefore can be positively correlated with the decision to register (selection effect, Λ > 1). On the other hand, it may also explain the difficulties of socializing with peers and lower self‐esteem (outcome effect, Γ > 1). Hence, the omission of this variable would mean that the estimated effect of registration on well‐being is overestimated. When turning to physical health outcomes, the individual's preference for health is one of the main potential confounders because this characteristic is likely to be positively correlated with registration (Λ > 1) and negatively correlated with the likelihood of experiencing an illness the last 4 weeks (Γ < 1), leading to an overestimation of the effect of registration on physical health.

**Figure 5:**
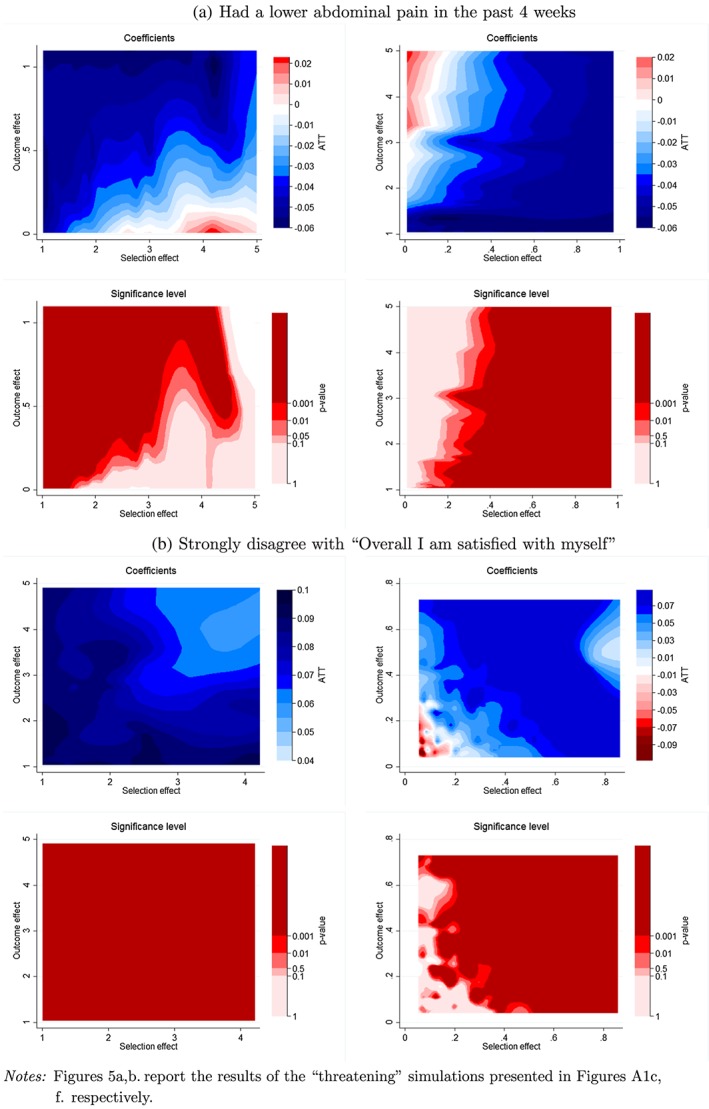
Killer confounders simulations. ATT: average treatment effect on the treated [Colour figure can be viewed at http://wileyonlinelibrary.com]

**Figure A1:**
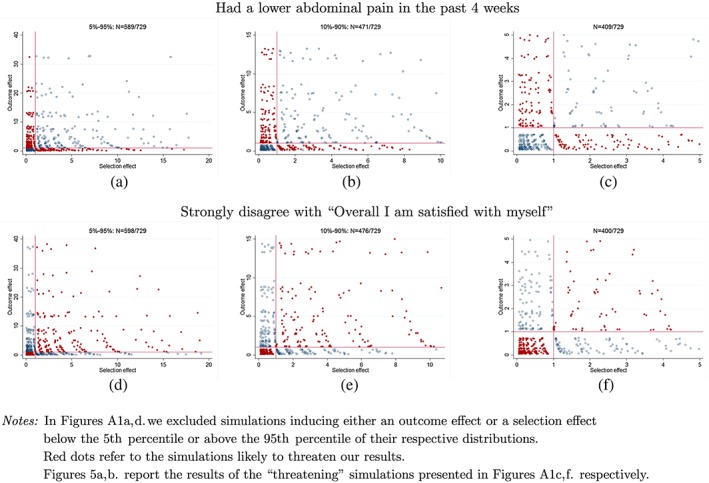
Simulations [Colour figure can be viewed at http://wileyonlinelibrary.com]

Two different exercises are implemented in this sensitivity analysis. In the first step, we simulate an unobserved variable, which would have similar selection and outcome effects than the one of covariates. Covariates with the greatest selection and outcome effects are reported in Table 5. We find that any unobserved variable with similar treatment and selection effects as the covariates already introduced in the propensity score matching will not confound our results. In the second step, we measure the size of the outcome and selection effect the unobserved variable should have in order to kill our results. To do so, we simulate all possible distributions of *U* (Figure A1 presented in Appendix A.2). Table 5 displays some examples of outcome and selection effects for which our main results would disappear. Lastly, Figure 5 presents the results of the sensitivity analysis. We show that in order to find a statistically insignificant effect of registration on health, the potential confounder should have an outcome effect and a selection effect that is 2 and 3 times greater respectively than what we observe in the covariates distribution. As for killing our results on well‐being, the outcome and selection effects should be almost 3 and 5 times bigger, respectively. The robustness of the results obtained in the main analysis are confirmed graphically in Figure 5 where we can note that the ATT and significance levels shrink only for high levels of selection and outcome effects.

**Table 5:** Sensitivity analysis

	Outcome	Selection		
	effect	effect		
	Γ	Λ	ATT	*SE*
Outcome: Had lower abdominal pain in the past month				
PSM result	—	—	*−0.055*	*0.031*
Confounder‐like				
Divorced	0.922	1.653	−0.054	0.005
Preference towards the future	0.700	1.488	−0.054	0.004
Has at least one child	1.609	0.908	−0.055	0.002
Entered the business alone	1.196	0.695	−0.055	0.005
Own house	1.020	0.428	−0.056	0.007
Killer confounder				
*U*= preference for health	0.432	4.790	−0.024	0.015
*U*	1.126	0.045	−0.050	0.031
Outcome: Strongly disagree with “Overall I am satisfied with myself”				
PSM result	—	—	*0.090*	*0.028*
Confounder‐like				
No education	1.417	1.736	0.088	0.005
Father or mother live in Dakar	0.762	0.531	0.088	0.005
Fatality	0.247	0.565	0.075	0.008
Own house	0.915	0.427	0.089	0.006
Killer confounder				
*U* = sexual abuse	4.323	8.846	0.034	0.022
*U*	0.023	0.371	0.027	0.017

*Note*. All covariates are binary variables. Five hundred replications have been performed for the sensitivity analysis. ATT: average treatment effect on the treated; PSM: propensity score matching.

### 5.4 Robustness checks

Propensity score matching is widely used in the literature to assess the causal effect of a program on a set of outcomes of interest, controlling for the biases induced by self‐selection into the program. Yet, besides CIA, consistency of the propensity score estimator relies also on correct specification and balancing property. We conduct two alternative methods to preprocess data to avoid these caveats: namely, entropy balancing to achieve balance in covariates beyond the mean (Appendix A.3) and a super learner to improve specification of the estimation of propensity scores (Appendix A.4). We show that the use of these additional methods that increase the validity of the propensity score estimator do lead to similar results. Finally, given the number of outcomes considered, we account for a false‐discovery rate to control for the proportion of type 1 error, that is, false rejection of the null hypothesis by performing the Benjamini–Hochberg (1995) correction and similar significance levels were found after correction. 8Results are available upon request from authors.


## 6 DISCUSSION

We analyzed the effects of the registration policy for FSWs in Senegal. Our results show that this policy is effective in inducing high‐risk populations into using services to prevent and treat STIs. However, the results also confirm that becoming a registered FSWs deteriorates well‐being.

Our results point out the need of psychological services to FSWs part of the registration policy. In addition, it shows that stigma attached to the current policy is counterproductive to HIV prevention efforts and prevents universal coverage. Because 56% of FSWs declare that the card is the main reason for not registering, it is important to explore ways to reduce the risks associated with the possession of the card. Over the last few years, the Ministry of Health have focused its efforts on changing the appearance of the registration card in order to make it more discreet and less stigmatizing, but this did not help increase registration, which points towards the need to remove the card. Although Senegal does not currently have the infrastructure to dematerialize the proof of registration through the use of an online database, e‐health and m‐health technologies could provide an effective low‐cost solution. For instance, health visits could be tracked on a mobile application or a quick response code valid for 1 month could be issued at each medical visit. Less radical interventions such as ones that would enable peers or bar/brothel owners to store the card could also be effective. In addition, FSWs' routine visits must be better integrated to maternal healthcare services. Compulsory monthly visits held on a specific day limits privacy and increases the risk of stigma. As a result, FSWs often decide to register in a health facility far from their home, increasing high direct and indirect costs, which acts as a barrier to registration.

Our study assumed that FSWs select themselves into either registration or illegality. Our main assumption is that the choice of status is determined by exogenous characteristics affecting the costs and the benefits of registration and as a result we assume that it is unlikely that FSWs switch status over time. We test this assumption by inviting participants to another survey that took place in August 2017, that is, 2 years after the survey that is used in this study. Among the 377 FSWs who could be contacted and who were still sex workers, only 26 (out of 208) became registered sex workers within this 2‐year time frame. Conversely, only three (out of 169) became unregistered in the same time frame. In addition, we conducted a willingness to accept exercise with unregistered FSWs, and 56% of participants refused the maximum hypothetical amount offered to register (CFAF 60,000), reflecting the difficulty to leverage registration over time due to the high stigma attached to this policy.

The conclusions of this study are based on data collected from registered and unregistered FSWs in Dakar. Despite providing the first evidence of the effect of compulsory registration for FSWs in Senegal, our study has some shortcomings. First, although the sample of registered FSWs is likely to be representative of this population, unregistered FSWs were recruited using snowball sampling, and this sample is likely to overrepresent unregistered FSWs who are connected to FSWs groups and nongovernmental organizations. In addition, we were not able to include under‐18 FSWs for ethical reasons. One may want to argue however that although not generalizable to the whole population of FSWs, our results still have good external validity. Indeed, because interventions to encourage registration will not reach the most isolated unregistered FSWs, the effects of the registration policy presented in this study are likely to be closer to the ones we would observe if an intervention to leverage registration was implemented in Senegal.

The main limitation of the paper is that it assumes that the registration policy only affects the intensive margins: Our paper investigates the effect of registration on the behaviors of the current pool of FSWs but falls short in investigating the effects at the market level. In fact, the effects of registration on market size and on the type of FSWs entering the sex work market are unknown. If registration attracts riskier FSWs, the policy could have detrimental effect on population health, even if the spread of STIs and HIV/AIDS is contained within the existing pool of FSWs. The effect of registration hence depends on context (e.g., quality of care, STI/HIV prevalence, and extent of an appeal of services to unregistered FSWs) and on the behavioral response of FSWs and clients.

Future research building on the results presented in this paper will consist in using mathematical and economic modeling to estimate the cost‐effectiveness of the registration policy in comparison with other HIV prevention policies (e.g., antiretroviral based prevention strategies) among FSWs in Senegal.

## 7 CONCLUSION

Few countries worldwide have opted for the regulation of sex work and mandatory medical screening of FSWs in order to control the spread of STIs and HIV. In Senegal since 1969, FSWs have to carry a registration card that contains information on the compliance to monthly health checks. Using primary data from registered and non‐registered FSWs, we investigate the effect of becoming a registered FSWs on physical health and well‐being. We show that in Senegal, becoming a registered FSW leads to a greater use of HIV/STI prevention services and reduced STI prevalence. However, because of the stigma attached to registration, we also show that this policy has a detrimental effect on emotional and social well‐being of registered FSWs. This later element suggests that changes in the registration policy are required in order to eliminate barriers to registration and their negative consequences on FSWs' well‐being.

## Figures and Tables

**Figure A2:**
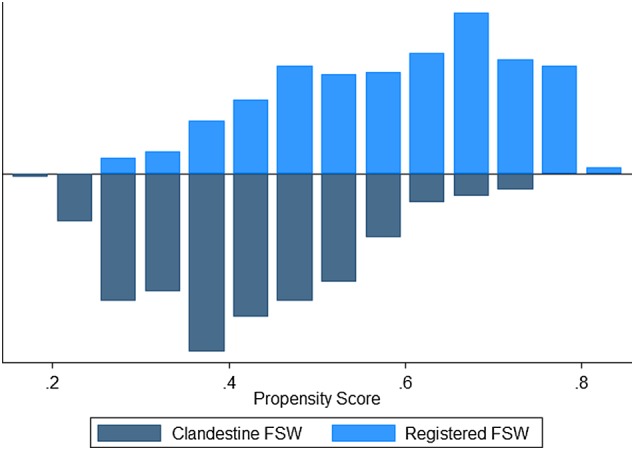
Common support based on super learner weights. FSW: female sex worker [Colour figure can be viewed at http://wileyonlinelibrary.com]

## APPENDIX A

### A.1 Model appendices

#### A.1.1 Full description of the setup

Let us consider a country where prostitution is regulated. Sex workers can, in such context, choose between two types of prostitution: either they choose to solicit clients in public places or they choose to remain discreet. If they choose to solicit clients in public places, they will access a larger pool of clients, but to do so, they need to register with authorities to avoid penal sanctions. Therefore, a sex worker chooses both the number of sex acts *a* and her registration status *R* = {0,1} in order to maximize her utility. Let us denote *a* ^0^ and *a* ^1^ the number of sex acts performed by an unregistered female sex worker (FSW) and a registered FSW, respectively.

Despite the legalization of prostitution by a government, prostitution is often morally condemned by the society. We assume that if identified by her relatives, friends, or neighbors, a sex worker suffers from *external* stigma *s*. We assume that external stigma depends on the level of wealth of the family, $s(A)>0,\forall A\in R_{+}$, as it may damage a family reputation. More precisely, a wealthier family may feel more strongly than less wealthy families that their reputation is tarnished if one of their members is known to be working as a sex worker. Furthermore, poorer families may be less contemptuous when discovering the source of the revenues the sex worker brings to the household if they are strongly financially constrained. As a result, FSWs from poorer families are less likely to be excluded from the household although they may still experience some external stigma within the household. We thus assume *s* *′*(*A*) > 0 for all *A*. In addition, a FSW may also suffer from damages of her self‐image and self‐esteem when engaging in commercial sex acts. We call this negative utility *internal* stigma, *τ*. We assume that internal stigma is nondecreasing in the number of sex acts, $\tau^{\prime }(a)⩾0$ for all *a*.

Registration is widely believed to increase the probability *δ* of being identified as a sex worker by friends, neighbors, or relatives. This is a direct consequence of multiple elements related to registration that are implemented in Senegal, such as holding a registration card issued only to FSWs, working in public places, and being registered in police files. Accordingly, we assume that, for a given *a*, the probability of being identified as a sex worker by others is larger for registered FSWs than for unregistered FSWs, $\delta^{1}⩾\delta^{0}$. 9 This assumption of differential probability may seem to be conjectural rather than factual. However, based on anecdotes from focus groups discussions in which they stressed a worry of the registration card to be found by relatives, we believe this assumption is realistic in the Senegalese context. Our data also show that the main reason for not registering is the preference for discreetness, which hints that FSWs believe $\delta^{1}⩾\delta^{0}$.
^,^ 10 We also note that predictions of the model are sharper if we do not impose it and use *δ* ^0^ = *δ* ^1^ or *δ* ^0^ > *δ* ^1^ instead, yet we can still derive the results with $\delta^{1}⩾\delta^{0}$.

Although the costs of registration are not clear cut, its benefits are unambiguous. If FSWs do not register with authorities and solicit clients in public places, they risk a prison sentence and/or a fine, which we denote collectively as a penalty *m* > 0. This penalty only applies to non‐registered FSWs. The penalty sanction is likely to be stochastic in nature, and its chance *r*(*a*) is nondecreasing in *a*, $r^{\prime }(a)⩾0$ for all *a*. Another benefit of registration is that registered FSWs receive a medical follow‐up that aims to prevent (through condom use distribution) and treat sexually transmitted infections (STIs), which is expected to have a positive effect on FSWs' health. We will introduce the health benefits of the registration program in a second step.

The utility of a FSW under registration status *R* depends on her consumption *c* ^*R*^, expected external stigma, internal stigma, and expected legal penalty. We assume consumption and various utility costs to be additively separable.

(A1) $$U=u(c^{R})-\delta^{R}(a^{R})s(A)-\tau (a^{R})-(1-R)r(a^{R})m.$$

We assume *u* is increasing and concave in *c* ( $u^{\prime }(c)>0,u^{\prime \prime }(c)⩽0,\forall c\in R_{++}$), the chances of being known and being arrested are both nondecreasing in *a* ( $\delta^{\prime \prime }(a)⩾0,r^{\prime }(a)⩾0$), internal stigma is nondecreasing in *a* ( $\tau^{\prime }(a)⩾0$), and external stigma is increasing in *A* (*s* *′*(*A*) > 0). 11 For completeness, we note that we assume *s*(0) > 0, that is, that even the poorest FSW feels external stigma if being identified as a sex worker. We further assume that *δ* and *r* follow symmetric, bell‐shaped density functions, which implies that the density is increasing for values below mean and decreasing for values above mean *μ*_*a*_, or $\delta^{R^{\prime \prime }}(a)⩾0$ for $a⩽\mu_{a}$, $\delta^{R^{\prime \prime }}(a)<0$ for otherwise, and $r^{\prime \prime }(a)⩾0$ for $a⩽\mu_{a}$, *r* *′* *′*(*a*) < 0 otherwise.

Next, we consider the market conditions. Given that the non‐registered FSWs cannot work in all venues and are limited to a subset of the market, 12 This is confirmed in our database, in which a larger share of non‐registered FSWs operates at home. we conjecture that the market size is bigger for the registered FSWs. So the (inverse) demand for registered and non‐registered FSWs differ, and the former is larger, that is, $w^{0}(a)⩽w^{1}(a)$. 13 In Section 3, we found that price distributions for registered and non‐registered FSWs overlap and their means are not statistically significantly different from each other. At the same time, the number of acts is larger for the registered FSWs, and their mean difference is statistically significant. The registered FSWs derive larger incomes from sex acts than the non‐registered FSWs. The strict inequality holds when a bigger registered market has clients who have higher willingness to pay for all sex act levels *a*. This implies that, for a given *a*, a marginal income of sex work is no smaller for the registered:

$$w^{1^{\prime }}(a)a+w^{1}(a)⩾w^{0^{\prime }}(a)a+w^{0}(a)\;\forall a\in R_{+}.$$

In addition, we assume that such a difference in market size is bounded from below. More precisely, we form a ratio of marginal incomes and assume that it is bounded from below at $\frac{\delta^{1^{\prime }}}{\delta^{0^{\prime }}}$: It is large enough that accessing a bigger market is beneficial even if it implies a greater chance of being identified as a sex worker, which is satisfied for $a⩽\mu_{a^{1}}$, where *μ*_*a*_ is mean of *a* under a symmetric, bell‐shaped density.

Assumption 2.1′ For all $a\in R_{+}$:

$$\frac{w^{1^{\prime }}(a)a+w^{1}(a)}{w^{0^{\prime }}(a)a+w^{0}(a)}⩾\frac{\delta^{1^{\prime }}(a)}{\delta^{0^{\prime }}(a)}.$$

The inequality in Assumption 2.1′ assumes that the ratio of marginal incomes is larger than the ratio of marginal probabilities. Intuitively, it states that, under registration, the marginal gain in income is greater than the marginal loss in terms of chances of being identified as a sex worker. When registration increases the probability of being known, or $\delta^{1}(a)⩾\delta^{0}(a)$ for all *a*, $\delta^{1^{\prime }}(a)>\delta^{0^{\prime }}(a)$ under Assumption 2.1′ is consistent with bell‐shaped density functions (under parallel displacement due to location parameter changes) for all $a⩽\mu_{a^{1}}$. Note also that it is independent of *A*, so any FSW will choose to supply more sex acts had they registered. This, of course, does not mean that all FSWs will be better off by registering and by supplying a larger number of sex acts.

#### A.1.2 The FSW's problem

A FSW's income is made of her sex work earning and other earnings such as assets, other occupation revenues, and transfers. We consider earnings outside sex work as exogenously given and summarize them as an asset *A*. A FSW under registration status *R* solves the following maximization program:

(A2) $$\begin{array}{ccc} \max_{\left\{a^{R}\right\}} & \;\;\;u(c^{R})-\delta^{R}(a^{R})s(A)-\tau (a^{R})-(1-R)r(a^{R})m & \\ s.t. & \;\;\;w^{R}(a^{R})a^{R}+A=c^{R}. \end{array}$$

The first order condition (FOC) is

(A3) $$F\equiv u^{\prime }\cdot (w^{R^{\prime }}a^{R}+w^{R})-\delta^{\prime \prime }(a^{R})s(A)-\tau^{\prime }(a^{R})-(1-R)r^{\prime }(a^{R})m=0.$$

A FSW chooses *a* ^*R*^ to equate the marginal consumptive utility with all marginal costs, expected external stigma, internal stigma, and an expected legal punishment if not registered (*R* = 0). As the marginal income is larger for the registered $w^{1^{\prime }}(a)a+w^{1}(a)⩾w^{0^{\prime }}(a)a+w^{0}(a)$, for a given *A*, Equation A3 immediately gives that $a^{1}⩾a^{0}$.

In Figure 1, the optimal *a* ^*R*^ is given by the intersection *e* ^*R*^ of *u* *′* and $\frac{\delta^{R^{\prime }}s+\tau^{\prime }+(1-R)r^{\prime }m}{w^{R^{\prime }}a^{R}+w^{R}}$.

To make sure that the solution is a maximizer, we assume the following (for more details on the concavity of the maximization problem, see Appendix A.1.4):

Assumption 2.2′ Internal stigma *τ* is an increasing, convex function of *a*, such that it dominates the decrease in density $\delta^{\prime \prime }$ and *r* *′*(*a*) for any level of *A* and *m* for a large *a*:

$$\delta^{R^{\prime \prime }}(a)s(A)+\tau^{\prime \prime }(a)+(1-R)r^{\prime \prime }(a)m⩾0.$$

For a small *a* under bell‐shaped density for $\delta^{\prime \prime }$ and *r* *′*, this is always satisfied.

A FSW will register if the value function under registration *V* ^1^ is larger than the one under illegality *V* ^0^. Because the FOC depends on *A* through marginal utility and marginal expected external stigma, the decision to register also depends on *A*. A FSW decides to register if

(A4) $$\begin{array}{ccc} V^{0}(A) & =u\left\{w\left(a^{0}\right)a^{0}+A\right\}-\delta^{0}\left(a^{0}\right)s(A)-\tau \left(a^{0}\right)-r\left(a^{0}\right)m & \\ & <u\left\{w^{1}\left(a^{1}\right)a^{1}+A\right\}-\delta^{1}\left(a^{1}\right)s(A)-\tau \left(a^{1}\right)=V^{1}(A). \end{array}$$

In other words, a FSW will register if the sum of extra consumptive utility, obtained from the increased supply of sex acts and the disappearance of expected legal penalty, is greater than the expected increased external and internal stigma. By using the envelope theorem, one can show that *V* ^*R*^ is increasing in *A* and the slope is greater for *V* ^0^ than *V* ^1^, because the marginal utility is greater and (negatively signed) probability of being known is no larger for the non‐registered FSWs. So the inequality (A4) is likely to hold for a small *A* (see Assumption 2.3′).

Assumption 2.3′ Equation 3 holds for a small enough *A*.

Given that external stigma depends positively on household wealth, one can show that the decision to register *R* switches from 0 to 1 as *A* becomes smaller. That is, there exists *A*_ such that *V* ^1^(*A*) > *V* ^0^(*A*) for *A* ≤ *A*_ as shown in Figure 2.

In a related manner, we note that a FSW with a smaller *A* provides more sex acts hence finds more benefits in registration. This is seen by deriving the following comparative statics result:

$$\frac{da^{R}}{dA}=-\frac{F_{A}}{F_{a}}=-\frac{u^{\prime \prime }\cdot (w^{R^{\prime }}a^{R}+w^{R})-\delta^{R^{\prime }}(a^{R})s^{\prime }(A)}{\text{SOC}}.$$

The denominator is the second order condition (SOC) $=\;u^{\prime \prime }\cdot {\left(w^{R^{\prime }}a^{R}+w^{R}\right)}^{2}+u^{\prime }\cdot (2w^{R^{\prime }}+w^{R^{\prime \prime }}a^{R})-\{\delta^{R^{\prime \prime }}s+\tau^{\prime \prime }(a)+(1-R)r^{\prime \prime }(a)m\}$ that holds strictly under the assumptions we have made (SOC =*F*_*a*_ < 0 ). Then, the above fraction has a negative sign: The poorer the FSW is, the more sex acts she will perform and the more likely she will decide to get registered.

We also note the effects of a penalty *m*. As it increases the marginal cost of supplying *a*, a larger *m* decreases the number of sex acts *a* ^0^ for the non‐registered FSWs. In Figure 2, one can see the effects of the introduction of a penalty *m* on the registration decision. More precisely, it shifts down the value function of non‐registration, changing the intersection to *b*_*m*_ and thus the associated threshold asset to a larger level denoted as *A*_(*m*) with *A*_*′*(*m*) > 0, *A*_(*m*) > *A*_(0) for $m\in R_{++}$. To sum up, a larger penalty induces FSWs with a larger *A* to register.

#### A.1.3 Health risk

We assume that a FSW is endowed with health capital, which gives the utility level of 0. For the number of sex acts chosen, there is a probability *π* that the FSW will be infected with an STI. If infected, the health utility will be reduced by *I*, which is the cost of illness. For simplicity, we assume that a FSW first chooses *a* and finds out her infection status after completing these sex acts. This ordering of events corresponds to the periodic timing of routine visits to the health center after supplying sex acts for some time for the registered, while we asume the same order of events for the non‐registered. We assume that an STI is a curable disease as in syphilis or a treatable disease as in the case of HIV/AIDS. We therefore let the health capital recover to the original level if treated.

The probability *π* of being infected is a function of the number of sex acts, with the chance nondecreasing in *a*, $\pi^{\prime }(a)⩾0$. All FSWs are assumed to face the same infection risk. 14 One may argue that clients' riskiness differs between registered and non‐registered FSWs. With a bigger market size for the registered, one may conjecture that there will be riskier clients, whereas one can also argue that the non‐registered will face the clients in the underground market, which may be riskier. Given there is no evidence on client's self‐selection process, we choose not to make a strong assumption on it. On the other hand, with a periodical medical follow‐up provided to registered FSWs, STI symptoms are more likely to be noticed and treated by health providers. We therefore assume that the probability of having an STI cured *β* ^*R*^ ∈ [0,1] is greater for registered FSWs than for unregistered FSWs, $\beta^{0}⩽\beta^{1}$. The net effect of registering on health is unambiguously beneficial for any level of *a*: $-\pi (a)(1-\beta^{0})I⩽-\pi (a)(1-\beta^{1})I$.

The health inclusive utility is given as *U* = *u* − *δ* *s* − *τ* − (1 − *R*)*r* *m* − *π*·(1 − *β* ^*R*^)*I*.

After introducing the health risk, we modify Assumptions 2.1′, 2.2′, and 2.3′ in the following way:

Assumption 2.1′ For all $a\in R_{+}$:

$$\frac{w^{1^{\prime }}(a)a+w^{1}(a)}{w^{0^{\prime }}(a)a+w^{0}(a)}⩾\frac{\alpha \delta^{1^{\prime }}(a)+(1-\alpha )\pi^{\prime }(a)(1-\beta^{1})}{\alpha \delta^{0^{\prime }}(a)+(1-\alpha )\pi^{\prime }(a)(1-\beta^{0})},\;with\;\alpha =\frac{s(A)}{s(A)+I}.$$

Assumption 2.2′ Internal stigma *τ* is an increasing, convex function of *a*, such that it dominates the decrease in density $\delta^{\prime \prime }$, *r* *′*(*a*), and *π* *′*(*a*) for any *A*, *m*, *β* ^*R*^, *I* for a large *a*:

$$\delta^{R^{\prime \prime }}(a)s(A)+\tau^{\prime \prime }(a)+(1-R)r^{\prime \prime }(a)m+\pi^{\prime \prime }(a)(1-\beta^{R})I⩾0.$$

For a small *a* under bell‐shaped density for $\delta^{\prime \prime }$ and *r* *′*, this is always satisfied.

Assumption 2.3′ Equation A8 holds for a small enough *A*.

Then we can derive the following:

Proposition 2.1′ There exists *A*__*I*_ such that $V^{1}(A)⩾V^{0}(A)$ for *A* ≤ *A*__*I*_: For a small enough level of wealth, a FSW decides to register, and ${\underline{A}}_{I}⩾\underline{A}$.

Proposition 2.2′
$a^{1}⩾a^{0}$ holds under the health risks. This holds even under *β* ^0^ = *β* ^1^. *a* ^*R*^ is smaller than in the absence of infection risk for *R* = {0,1}.

Proposition 2.3′ The greater the probability *β* ^*R*^ of being cured, the greater the sex act supply *a* ^*R*^. The access to quality health services increases the number of sex acts.

Proposition 2.4′ The greater the damage *I*, the smaller the sex act supply *a* ^*R*^. The severity of a disease reduces the number of sex acts.

Remark 2.1′ If the relative risk of being infected for registered FSWs compared with non‐registered FSWs is lower (greater) than the relative probability of being treated, that is, $\frac{\pi (a^{1})}{\pi (a^{0})}<(>)\frac{\left(1-\beta^{0}\right)}{\left(1-\beta^{1}\right)}$, registration ensures a lower (greater) expected physical health damage.

Proposition 2.1′ shows that the threshold asset level of registration is larger than in the absence of infection risk due to the relative curative effectiveness under registration. Proposition 2.2′ shows that registered FSWs still work more intensively than unregistered FSWs even if this leads to greater infection risks. It shows that the prospect of health damage and subsequent access to health services for registered FSW make registration more attractive. It also shows that, even if there is no difference in the probability of being treated, *β* ^0^ = *β* ^1^, it is possible for a FSW to register and supply larger *a* relative to non‐registered status. This can pose a challenge to public health because the non‐health merits of registration induce FSWs to register, under which they supply more *a* and get infected more frequently, and unless they have a superior cure rate, it translates to higher STI incidence. Proposition 2.3′ reflects the moral hazard resulting from a higher probability *β* ^1^ of being cured when clinics cannot observe risky acts. In fact, if the probability of being cured is 1, the supply of sex act will no longer be affected by infection risk and the maximization problem (A5) is reduced to (A2). Proposition 2.4′ expresses that there is a negative relationship between the severity of STIs that FSWs could contract and the number of sex acts they supply. Finally, Proposition 2.2′ indicates $\pi (a^{1})⩾\pi (a^{0})$ while we assume $1-\beta^{0}⩾1-\beta^{1}$. Therefore, Remark 2.1′ indicates that the expected health damage may or may not increase after registration $\pi (a^{1})(1-\beta^{1})⋚\pi (a^{0})(1-\beta^{0})$. The effect of registration on health outcomes is ambiguous and will depend on the extent of increase in infection risks *π*(*a* ^1^) − *π*(*a* ^0^).

#### A.1.4 Concavity of the maximization problem

SOC for the maximum under (p1) is given as below:

$$F_{a}=u^{\prime \prime }\cdot {\left(w^{R^{\prime }}a^{R}+w^{R}\right)}^{2}+u^{\prime }\cdot (2w^{R^{\prime }}+w^{R^{\prime \prime }}a^{R})-\{\delta^{R^{\prime \prime }}(a)s(A)+\tau^{\prime \prime }(a)+(1-R)r^{\prime \prime }(a)m\}<0.$$

The SOC holds if marginal income is nonincreasing ( $2w^{R^{\prime }}+w^{R^{\prime \prime }}a^{R}⩽0$, which holds for non‐Giffen goods), and probability *δ* ^*R*^ is convex at *a* ^*R*^ ( $\delta^{R^{\prime \prime }}(a^{R})⩾0$) and *τ* *′* *′*(*a* ^0^) is convex at *a* ^0^, both of which hold for a smooth, bell‐shaped density at $a^{R}⩽\mu_{a^{R}}$. For $a^{R}>\mu_{a^{R}}$, it is possible that, given that *δ* ^*R*^(*a*),*r*(*a*) are already high enough, the marginal increase in these probabilities can become smaller as one increases *a*, which is the case for bell‐shaped density functions with an infinite support. However, this induces the FSWs to supply as much sex acts as possible, which is clearly unrealistic. So we assume the following for SOC to hold:

Assumption 2.2:

$$\delta^{R^{\prime \prime }}(a)s(A)+\tau^{\prime \prime }(a)+(1-R)r^{\prime \prime }(a)m⩾0.$$

Assumption 2.2 is a sufficient condition for the SOC for the maximum to hold at a large *a*. For $a^{R}>\mu_{a^{R}}$, we have decreasing marginal density, and it is necessary to assume that the marginal increase of internal stigma is large enough to ensure the SOC to hold. The convexity assumption of internal stigma is consistent with our data in which more active FSWs report more psychological problems.

#### A.1.5 Comparative statics with health

A FSW under registration status *R* now solves the following maximization program:

(A5) $$\begin{array}{ccc} \max_{\left\{a^{R}\right\}} & \;\;\;u(c^{R})-\delta^{R}(a^{R})s(A)-\tau (a^{R})-(1-R)r(a^{R})m-\pi (a^{R})(1-\beta^{R})I & \\ s.t. & \;\;\;w^{R}(a^{R})a^{R}+A=c^{R}. \end{array}$$

The FOC for (A5) is

(A6) $$F_{2}\equiv u^{\prime }\cdot (w^{R^{\prime }}a^{R}+w^{R})-\delta^{\prime \prime }(a^{R})s(A)-\tau^{\prime }(a^{R})-(1-R)r^{\prime }(a^{R})m-\pi^{\prime }(a^{R})(1-\beta^{R})I=0.$$

Compared with the FOC (2) in the absence of infection risks, there is an extra negative term −*π* *′*(*a* ^*R*^)(1 − *β* ^*R*^)*I*, which makes *a* ^*R*^ to be smaller.

Again, to ensure that the solution is the maximum, we modify Assumption 2.2 as Assumption 2.2′. Assumption 2.3′ holds for a larger *A* than in Equation 3, so we can find *A*__*I*_ in an analogous way as in Proposition 2.1, only that ${\underline{A}}_{I}⩾\underline{A}$ because the expected infection damage is larger for the non‐registered FSWs.

We can derive how the number of acts *a* ^*R*^ varies with wealth *A*, with the probability of being cured *β* ^*R*^ and with the size of STI damage when left untreated *I* with SOC denoted as F_2a_which holds under Assumption 2.2′.

(A7) $$\begin{array}{ccc} \frac{da^{R}}{dA} & =-\frac{u^{\prime \prime }\cdot (w^{R^{\prime }}a^{R}+w^{R})-\delta^{R^{\prime }}(a^{R})s^{\prime }(A)}{F_{2a}}⩽0, & \\ \frac{da^{R}}{d\beta^{R}} & =-\frac{\pi^{R^{\prime }}(a^{R})I}{F_{2a}}⩾0,\\ \frac{da^{R}}{dI} & =\frac{\pi^{R^{\prime }}(a^{R})(1-\beta^{R})}{F_{2a}}⩽0. \end{array}$$

Again, a FSW decides to register if her maximized utility under registration *V* ^1^ is greater than under illegality *V* ^0^:

(A8) $$\begin{array}{ccc} V^{0} & =u(c^{0})-\delta^{0}(a^{0})s(A)-\tau (a^{0})-r(a^{0})m-\pi (a^{0})(1-\beta^{0})I & \\ & <u(c^{1})-\delta^{1}(a^{1})s(A)-\tau (a^{1})-\pi (a^{1})(1-\beta^{1})I=V^{1}. \end{array}$$

In other words, a FSW would register if the increase in consumptive utility, decrease in health risks, and averted expected penalty amount is greater than the increased external and internal stigma costs. This holds for a larger *A* than in Equation 3, so we can find *A*_ in an analogous way as in Proposition 2.1.

### A.3 Robustness check: Improving covariates balance using entropy balancing

Hainmueller (2012) introduced entropy balancing, a data preprocessing procedure that directly focuses on covariate balancing and is able to ensure balancing not only on the first moment of the distribution but also on any specified moment. One can then force the distribution of all covariates considered to look very similar in the treatment and in the reweighted control groups, thereby simulating a randomized experiment. We implement entropy balancing so that the three first moments of the distribution of each covariate used to estimate the propensity score are identical for registered and non‐registered FSWs. Table A1 shows the differences between the two groups before and after implementing the entropy balancing procedure. Weights generated by this procedure are then used to estimate the causal effect of registration on the outcomes of interest, which lead to similar results as the ones obtained with propensity score matching (see Tables A3 and A4).

**Table A1:** Balancing of covariates

	Means			Variances			Skewness		
	Controls			Controls			Controls	
Covariates	Pre	Post	Treated	Pre	Post	Treated	Pre	Post	Treated
Age	36.06	36.81	36.81	82.49	75.01	75.01	0.301	0.284	0.284
Divorced	0.670	0.745	0.745	0.222	0.191	0.190	−0.723	−1.126	−1.126
No education	0.227	0.330	0.330	0.176	0.222	0.222	1.307	0.722	0.722
Has at least one child	0.896	0.881	0.881	0.093	0.106	0.106	−2.602	−2.346	−2.346
Father/mother live in Dakar	0.625	0.472	0.472	0.235	0.250	0.250	−0.515	0.113	0.113
Preference towards the future	0.178	0.233	0.233	0.147	0.179	0.179	1.684	1.265	1.265
Altruism (more than 400 FCFA)	0.197	0.387	0.387	0.159	0.238	0.238	1.520	0.465	0.465
Risk aversion	0.725	0.709	0.709	0.571	0.587	0.587	0.755	0.775	0.775
Beauty	0.799	0.745	0.745	0.161	0.190	0.190	−1.495	−1.126	−1.126
Entered the sex business alone	0.576	0.487	0.487	0.245	0.251	0.251	−0.308	0.050	0.050
Fatality	0.702	0.572	0.572	0.210	0.246	0.246	−0.885	−0.293	−0.292
Own house	0.269	0.135	0.135	0.197	0.117	0.117	1.044	2.132	2.133

### A.4 Robustness check: Improving specification of the propensity score using a super learner

Although it is common practice to use logistic regression to estimate propensity score, there is evidence that propensity score model misspecification can lead to biases in treatment estimation (Drake, 1993). Machine learning methods can be used for propensity score estimation in order to choose the optimal regression algorithm among a set of candidates (Pirracchio, Petersen, & van der Laan, 2014). We implement the method proposed by van der Laan, Polley, and Hubbard (2007) so that a weighted linear combination of the candidate learners associated with a high performance is used as a super learner estimator. To achieve this, we include 15 different models in the super learner library (see Table A2). Table A2 displays the composition of the super learner estimator. Figure A2 shows the common support based on the super learner propensity score estimation. We can note that the overlap is reduced compared with before (see Figure 4) but that the distributions in the treatment and control groups are more asymmetric. Despite having a lower number of units on support (563 vs. 626), ATTs based on the scores estimated with a super learner remain of comparable magnitude (see Tables A3 and A4) than the ones obtained using a logistic regression.

**Table A2:** Composition of the super learner

Models	Risk	Coefficient
Stepwise regression with interactions	0.2425	0.2248
Logistic regression	0.2340	0.0000
Generalized additive model	0.2343	0.0000
Random forest	0.2375	0.0482
Polynomial spline regression	0.2425	0.0000
Neural network	0.2537	0.0360
Stepwise regression	0.2393	0.0000
Bayesian generalized linear model	0.2338	0.2786
Classification and regression routines	0.2371	0.0116
Classification and regression trees (CART): Recursive partitioning	0.2512	0.0000
Bootstrapped aggregated CART	0.2346	0.0000
Gradient boosting	0.2315	0.1508
Support vector machine	0.2381	0.2500
Generalized linear model with penalized maximum likelihood	0.2334	0.0000
Multivariate adaptive regression splines	0.2406	0.0000

*Note*. A low risk indicates a greater performance of the model. The coefficients indicate how much weight the super learner puts on each model in the weighted‐average.

**Table A3:** Robustness checks—Final outcomes

			Entropy		Super	
	PSM		balancing		learner	
Outcomes	*ATT*	*Sign.*	Coeff.	Sign.	ATT	Sign.
Observations	T = 318	C = 308			T = 280	C = 283
Physical health						
Has been sick or injured in the past 4 weeks	−0.109	^∗∗^	−0.120	^∗∗∗^	−0.133	^∗∗^
Had lower abdominal pain in the past month	−0.055	^∗^	−0.060	^∗^	−0.047	NS
Well‐being						
Is not happy	0.077	^∗^	0.086	^∗∗^	0.074	NS
Is not satisfied at all in general	0.074	^∗∗^	0.074	^∗^	0.022	NS
Strongly disagree with						
“Overall I am satisfied with myself”	0.090	^∗∗∗^	0.093	^∗∗∗^	0.087	^∗∗∗^

*Note*. NS stands for “not significant.” Results in italic come from Table 3. ATT stands for average treatment effect on the treated. Results of the Gaussian Kernel matching on the common support with replacement and 1,000 replications are presented for super learner. T and C indicate the number of treated and control observations, respectively. Super learner propensity score is a weighted linear combination of candidates presented in Table A2.*p*< 0.1*p*< 0.05*p*< 0.01.

**Table A4:** Robustness checks—Intermediate outcomes

			Entropy		Super	
	PSM		balancing		learner	
Outcomes	*ATT*	*Sign.*	Coeff.	Sign.	ATT	Sign.
Observations	T = 318	C = 308			T = 280	C = 283
Prevention						
Received free condoms	0.369	^∗∗∗^	0.373	^∗∗∗^	0.324	^∗∗∗^
Is affiliated to a STD center	0.364	^∗∗∗^	0.358	^∗∗∗^	0.347	^∗∗∗^
Went to a health center in the last month	0.560	^∗∗∗^	0.554	^∗∗∗^	0.543	^∗∗∗^
Had a HIV screening in the past year	0.237	^∗∗∗^	0.221	^∗∗∗^	0.240	^∗∗∗^
Sought care last STI	0.025	NS	−0.041	NS	−0.045	NS
Sought care last illness	0.055	NS	0.065	NS	0.058	NS
Unhealthy behaviors						
Cigarette expenses in the last 7 days	560	^∗^	483	NS	704	^∗∗^
Alcohol expenses in the last 7 days	1300	^∗∗∗^	1335	^∗∗∗^	1498	^∗∗∗^
Sex work environment						
Number of clients per week	2.665	^∗∗∗^	2.617	^∗∗∗^	2.408	^∗∗∗^
Attracts usually clients in bars or nightclubs	0.338	^∗∗∗^	0.320	^∗∗∗^	0.362	^∗∗∗^
Last client was an occasional client	0.153	^∗∗∗^	0.151	^∗∗∗^	0.179	^∗∗∗^
Had alcohol before last sex act	0.062	^∗∗^	0.064	^∗∗∗^	0.061	^∗^
Last client consumed alcohol	0.125	^∗∗∗^	0.118	^∗∗∗^	0.135	^∗∗∗^
Multiple clients relationship during last sex act	0.051	^∗∗^	0.050	^∗∗^	0.052	^∗^
Used a condom during last sex act	0.016	NS	0.019	NS	0.003	NS
Anal intercourse during last sex act	0.019	NS	0.023	^∗∗^	0.025	^∗∗^
Fellatio during last sex act	0.015	NS	0.019	NS	0.024	NS
Is not satisfied at all with sex work	0.102	^∗∗^	0.087	^∗^	0.035	NS
In the past year:						
Suffered from violence of an occasional client	0.101	^∗^	0.063	NS	0.115	^∗^
Suffered from police violence	0.047	^∗^	0.068	^∗∗∗^	0.032	NS
Fear police						
In the past year:						
If suffered from client violence						
went to report it to the police	0.192	^∗∗^	0.103	NS	0.055	NS
Earnings and savings						
Monthly earnings from sex work (FCFA)	62793	^∗∗∗^	56792	^∗∗∗^	61930	^∗∗∗^
Savings in the past month (FCFA)	24332	^∗∗∗^	25232	^∗∗∗^	27559	^∗∗∗^
Leaving sex work						
Is sure that she will no longer						
be a FSW in 3 years	−0.066	NS	−0.082	^∗^	−0.065	NS
Social network						
Rivalry	54	^∗∗^	46	NS	−3	NS
Altruism towards other sexworker	2	NS	−1	NS	−1	NS
Life satisfaction with friends	−0.255	^∗∗∗^	−0.262	^∗∗∗^	−0.224	^∗∗∗^
Has at least one FSW to go to be reassured	−0.146	^∗∗∗^	−0.144	^∗∗∗^	−0.145	^∗^
Has at least one FSW to borrow money to	−0.122	^∗∗∗^	−0.124	^∗∗∗^	−0.135	^∗∗^
Stigma						
Family knows about her sex work activity	0.080	^∗∗^	0.069	NS	0.080	NS
Life satisfaction with family	−0.219	^∗∗∗^	−0.225	^∗∗∗^	−0.178	^∗∗^

*Note*. NS stands for “not significant.” Results in italic come from Table 4. ATT stands for average treatment effect on the treated. Results of the Gaussian Kernel matching on the common support with replacement and 1,000 replications are presented for super learner. T and C indicate the number of treated and control observations, respectively. Super learner propensity score is a weighted linear combination of candidates presented in Table A2. FSW: female sex worker; STI: sexually transmitted infection.*p*< 0.1.*p*< 0.05.*p*< 0.01.
